# Why, When and How to Adjust Your P Values?

**DOI:** 10.22074/cellj.2019.5992

**Published:** 2018-08-07

**Authors:** Mohieddin Jafari, Naser Ansari-Pour

**Affiliations:** 1 Drug Design and Bioinformatics Unit, Medical Biotechnology Department, Biotechnology Research Center, Pasteur Institute of Iran, Tehran, Iran; 2 Faculty of New Sciences and Technologies, University of Tehran, Tehran, Iran

**Keywords:** Bias, Gene Expression Profiling, Genetic Variation, Research Design, Statistical Data Analyses Cell Journal(Yakhteh), Vol 20, No 4, Jan-Mar (Winter) 2019, Pages: 604-607

## Abstract

Currently, numerous papers are published reporting analysis of biological data at different omics levels by making statistical inferences. Of note, many studies, as those published in this Journal, report association of gene(s) at the genomic and transcriptomic levels by undertaking appropriate statistical tests. For instance, genotype, allele or haplotype frequencies at the genomic level or normalized expression levels at the transcriptomic level are compared between the case and control groups using the Chi-square/Fisher’s exact test or independent (i.e. two-sampled) t-test respectively, with this culminating into a single numeric, namely the P value (or the degree of the false positive rate), which is used to make or break the outcome of the association test. This approach has flaws but nevertheless remains a standard and convenient approach in association studies. However, what becomes a critical issue is that the same cut-off is used when ‘multiple’ tests are undertaken on the same case-control (or any pairwise) comparison. Here, in brevity, we present what the P value represents, and why and when it should be adjusted. We also show, with worked examples, how to adjust P values for multiple testing in the R environment for statistical computing (http://www.R-project.org).

Biological data is currently being generated on a massive scale, which has resulted not only in an avalanche of raw data, but has also led to the testing of multiple hypotheses. To test these hypotheses, inferential statistics is applied to relevant sample datasets, leading to further biological insights and possible discoveries. Essentially, hypothesis testing is a statistical method which computes the probability of the strength of evidence based on the sampled data for or against the null (i.e. no difference or no change) hypothesis, which is culminated in a single numeric, namely the P value. Here, we discuss P values, but more importantly, with a focus on association studies, discuss why, when and how they should be adjusted. We hope that this short guide results in more accurate reporting of P values and the respective inferences.

## What is a P value?

When you want to statistically infer whether a result is significant, you quantify the probability of that result occurring by pure random chance given the null hypothesis. A historical and intuitive cut-off to reject the null hypothesis (thus a meaningful non-random event) is 0.05 (1). Accordingly, if the probability of testing the null hypothesis of equality of the mean of normalized expression levels of gene X in the case and control groups (µ_1_, µ_2_) is <0.05, one would say (absolutely arbitrarily) that it is their eureka moment by shrugging off (reject) the null hypothesis (µ_1_=µ_2_), and embracing (accept) the alternative hypothesis (µ_1_.µ_2_). However, what we are actually quantifying is the probability of observing data as or more extreme than what we have observed given the null hypothesis is true (2-4). Meanwhile, it should be noted that in statistical hypothesis testing, we should not only report the P value, but to also include power of test, confidence intervals and effect size (5-8). 

## P value issues

There is a matter of considerable controversy surrounding the position of Pvalue in scientific inference and this has become even more heightened by the emergence of big data analysis, which mainly revolves around its misunderstanding and misuse (9, 10). The first flaw is that the 0.05 cut-off is completely arbitrary and merely a convention. This, therefore, indicates that this value is not necessarily appropriate for all variables and for all research settings. For instance, in disease association studies, a more stringent cut-off of 0.01 is recommended to be applied. Moreover, two common biases further affect the integrity of research findings, namely selective reporting and P-hacking (7). In brief, selective reporting addresses the bias of substantially under-reported negative results (i.e. non-significant P values). This bias is apparent in the skewed distribution of reported results toward positive findings (11). In contrast, P-hacking describes the biased selection of data to signify non-significant results when this is desirable. Although this is technically true, it is a far more unrepresented form of direct data manipulation (12). 

## The multiple testing issue 

Assuming that all the flaws mentioned are addressed, the last but the most important issue that remains in P value quantification is when multiple testing occurs, but what constitutes multiplicity? Imagine a scenario where the expression of twenty genes at the transcript level have been compared between a fixed set of cases and controls or, at the genomic level, genotype/allele frequencies of twenty single nucleotide polymorphisms (SNPs) have been compared. By pure chance, assuming independence of tests, one would expect, on average, one in twenty of transcripts or SNPs to appear significant at the 5% level. This is because the ‘probability’ of a false positive in this scenario is now inflated and clearly requires adjusting the original single test significance level of 0.05. In other words, the probability of observing a false positive (i.e. type I error) generated by all tests undertaken should not exceed the 5% level (2). This issue has become ever more apparent after the emergence of omics science, in which large number of independent variables are tested simultaneously and computing the fraction of true positives is crucial (5). As a simple calculation, suppose the probability of a type I error in a single test is α_single_=5×10^-2^. The probability of not observing a type I error in a single test is then p_single_=1-α=1-5×10-2=0.95. Accordingly, the probability of not observing a type I error in multiple (e.g. 20) tests is p_multiple_=(1-α)m=(1- 5×10-2)20≈3.6e-01 and thus α_multiple_=1-(1-α)m ≈ 0.64, therefore showing the substantial increase in type I error after multiple testing. If the number of tests increases dramatically, the inflated type I error rate (α_multiple_) would reach 1. For instance, α_multiple_= 0.9941 if α=0.05 and m=100.

So how one ought to correct this inflation of the false positive rate? The first solution is to control type I error by minimising the significance threshold (i.e. calculating α’). Say the probability of a type I error in a single test is the standard α_single_=α´. The probability of not observing a type I error in a single test is then p_single_=1-α´. For independent tests, this probability would be p_multiple_=(1-α´)m. Next, the probability of type I error for multiple tests is α_multiple_=1-(1-α´)m. Rearrangment of the equation leads to the approximated Bonferroni correction for multiple testing α´≈ α/m. Following the same scenario, the α´ for each of the twenty tests would be 0.05/20=2.5×10-3. By applying the same rule, when 1,000,000 SNPs are tested in a genome-wide association study (GWAS) αˊ would be 5×10-8 and when expression dysregulation is examined for 20,000 genes on a whole-transcriptome microarray, αˊ would be 2.5×10^-6^. 

## How to adjust P values?

Here we provide worked examples for the two most commonly used methods without in-depth mathematical detail and formulae. This approach is analytically more convenient compared with the first method, in which, after setting an adjusted threshold, raw P values have to be checked against a' one at a time. The function used here is p.adjust from the stats package in R. Imagine you have tested the level of gene dysregulation between two groups (e.g. cases and controls) for ten genes at the transcript level and below is the vector of raw P values obtained by implementing the independent t test (assuming normality of expression data).

P_value <- c(0.0001, 0.001, 0.006, 0.03, 0.095, 0.117, 0.234, 0.552, 0.751, 0.985).

## Bonferroni

The simplest way to adjust your P values is to use the conservative Bonferroni correction method which multiplies the raw P values by the number of tests m (i.e. length of the vector P_values). Using the p.adjust function and the ‘method’ argument set to "bonferroni", we get a vector of same length but with adjusted P values. This adjustment approach corrects according to the family-wise error rate of at least one false positive (FamilywiseErrorRate (FWER)=Probability (FalsePositive≥1)). 

p.adjust (P_values, method="bonferroni") ## [1] 0.001 0.010 0.060 0.300 0.950 1.000 1.000 1.000 1.000 1.000 

The results show that only two out of ten genes remain significantly dysregulated. Had we not undertaken this multiple testing correction, we would have reported significant dysregulation for another two genes. This correction method is the most conservative of all and due to its strict filtering, potentially increases the false negative rate (5) which simply means rejecting true positives among false positives. 

## Benjamini and Hochberg

A philosophically different and more powerful adjustment method is that proposed by Benjamini and Hochberg (13). This method, rather than controlling the false positive rate (a.k.a FWER) as in the Bonferroni method, controls the false discovery rate (FalseDiscoveryRate (FDR)=Expected (FalsePositive/ (FalsePositive+TruePositive))). In other words, FDR is the expected proportion of false positives among all positives which rejected the null hypothesis and not among all the tests undertaken. In the FDR method, P values are ranked in an ascending array and multiplied by m/k where k is the position of a P value in the sorted vector and m is the number of independent tests. 

p.adjust (P_values, method="fdr") 

 ## [1] 0.001 0.005 0.02 0.075 0.19 0.195

## [7] 0.334 0.690 0.834 0.985 

A quick comparison of the results show that FDR identifies one more dysregulated gene compared with the Bonferroni method. This third gene (corrected P=0.02) is what would be called a false negative as it shows no significance when the conservative Bonferroni method is used but remains significant under FDR. 

**Fig.1:**
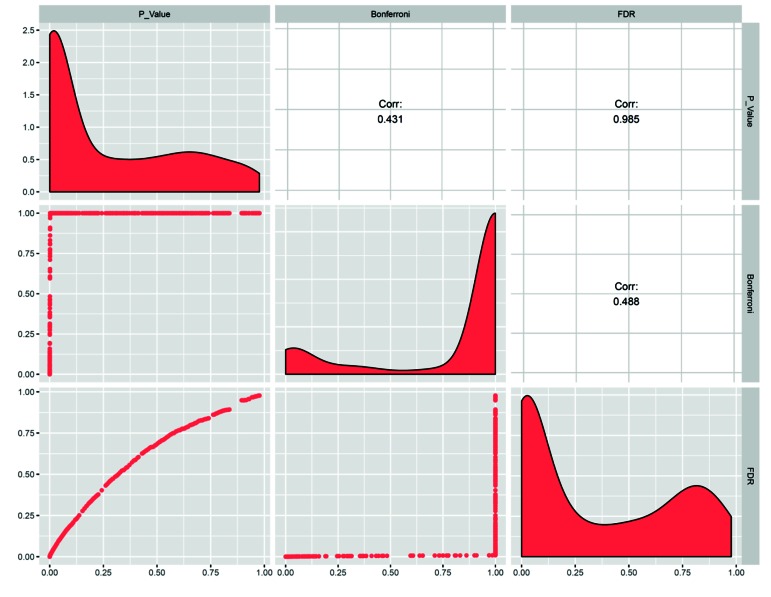
Comparison of the two multiple testing adjustment methods in a matrix plot. The distribution of 500 random P values before and after adjustment is represented on the diagonal. The upper and lower triangles show the pairwise correlation coefficients and scatter plot between raw and adjusted P values respectively.

To better compare these two multiple testing correction methods, a large array of random P values (n=500) were adjusted (Fig .1). The frequency distributions show that the Bonferroni method dramatically reduces the number of significant P values and substantially increases large (close or equal to 1) P values. However, the FDR method retains more significant P values while increasing non- significant P values with a peak at around P=0.8. This is consistent with a higher correlation between raw and FDR-adjusted P values than any other pairwise combination. Although a number of different multiple testing correction methods exists (for instance see p.adjust documentation in R or permutation-based correction methods), the most preferable approach is controlling FDR as it not only reduces false positives, but also minimises false negatives. 

The take home message is that it does not matter whether you are interested in identifying a significant association with SNPs, differentially expressed genes (DEG) or enriched gene ontology (GO) terms, the moment you conduct multiple tests on the same samples or gene sets respectively, it would be essential to address the multiple testing issue by adjusting the overall false positive rate through calculating a´ or adjusting your raw P values (as shown here based on Bonferroni or FDR) for true positives to be teased out. This will in no doubt enhance reliability and reproducibility of research findings.
